# A Novel Stable Binary BeB_2_ phase

**DOI:** 10.1038/srep06993

**Published:** 2014-11-11

**Authors:** Changzeng Fan, Ye Jin, Jian Li, Xu Dong

**Affiliations:** 1 State Key Laboratory of Metastable Materials Science and Technology, Yanshan University, Qinhuangdao 066004, China

## Abstract

Potential crystal structures of BeB_2_ were explored using *ab initio* evolutionary simulations. A new phase with a *Cmcm* space group was uncovered. It was determined that the *Cmcm* phase is mechanically and dynamically stable and has a lower enthalpy, from ambient pressure up to 13 GPa, than any previously proposed phases, as measured using first-principles calculations. The crystal structure, phonon dispersion, phase transitions, and mechanical and electronic properties of this phase were investigated. It was determined that the *Cmcm* phase may transform into the 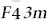 phase at pressures higher than 13 GPa. The band structures and density of states reveal that the *Cmcm* phase is metallic. In addition, the Vickers hardness was calculated using three empirical models. To explain the origin of the hardness, charge density difference maps and a Mulliken population analysis were carried out, which demonstrated that there are strong covalent interactions between B atoms. By analyzing the Crystal Orbital Hamilton Population (COHP) diagrams, it was determined that the total interaction of the Be-B bonds is stronger than that of the B-B bonds, indicating a very complex bonding feature in the new phase. It was predicted that the new *Cmcm* phase is nearly absent of superconductivity.

Beryllium (Be) and boron (B) are elements that occupy adjacent positions in the periodic table, but they have quite different properties, as the former is a metal while the latter is a semiconductor under standard conditions. At ambient conditions, Be crystallizes in a hexagonal-closed-packed (hcp) structure and transforms into a body centered cubic (bcc) crystal structure at 1523 K or approximately 400 GPa^1^,^2^. It has gained considerable attention because of its simple atomic configuration and unusual behaviors. As it is highly transmissive to X-rays, Be is commonly used as a window material in X-ray equipment^3^. It is also one of the most effective moderators and reflectors for neutrons used in nuclear reactors^4^. In addition, its light weight, high melting point and high-strength performance result in it being widely used in aircraft, rockets, satellites and other aerospace applications, as well as in future fusion reactors^5^,^6^. Although Be has several negative characteristics, such as potential toxicity that poses a manufacturing challenge, controlled handling procedures, such as the use of a glove box, can enable the success industrial synthesis of beryllium-containing compounds^7^,^8^.

Boron is a semiconductor that becomes a metal, and even a superconductor, when placed under high pressure^9^. Elemental boron has many allotropes that use the B_12_ icosahedron as the basic unit, of which α-Boron is the most common^10^. Boron-containing compounds have been investigated extensively due to their exotic physical properties, such as superconductivity, hardness and thermoelectric performance. For example, several transition metal diborides, such as OsB_2_, IrB_2_, ReB_2_ and BC_x_, possess high bulk and shear moduli and have been explored for use as potential super-hard materials^11^,^12^,^13^,^14^,^15^,^16^.

When beryllium and boron combine, various Be-B phases ranging from beryllium-rich to boron-rich will form. Some Be-rich phases, such as the Be_2_B phase, show a metallic character and are potential superconductors^17^. Another beryllium-rich phase, Be_4_B, is the only stable Be-rich phase at room temperature. Within the boron-rich compounds, BeB_3_, found only in the MgIn_3_ type (space group 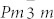, NO.221, one formula unit per cell), is found to be not stable. BeB_4_, however, is highly stable at high pressures^2^.

Although beryllium diboride has been known and studied for more than half century^18^,^19^,^20^,^21^,^22^,^23^, there has still been no solid evidence that it exists with the stoichiometry BeB_2_. The first announced synthesis of BeB_2.01 ± 0.03_ with *P6/mmm* symmetry^18^ was later recognized to have a correct stoichiometry of BeB_2.75_ with a surprisingly complex crystal structure^19^. Very recently, Hermann, A. *et* *al*. systematically studied the binary Be-B phases at ambient and high-pressure conditions^2^,^20^. In their work, they found that the most stable structure for BeB_2_ at atmospheric pressure is cubic (space group 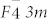, NO.216), with a diamondoid boron network and beryllium atoms occupying interstitial tetrahedral sites. This structure is semiconducting and can be understood in terms of the Zintl-Klemm concept, making it unique among Be-B phases. Other proposed beryllium di-boron phases can be classified into three crystal systems: (1) *cubic*, including the CaAl_2_ structure (space group 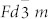, NO.227, eight units per cell, prototype MgCu_2_^24^ ) and “AlLiSi” structures (space group 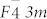, NO.216^20^); (2) *hexagonal*, including the MgB_2_ crystal structure (space group *P6/mmm*, NO.191^20^, one unit per cell, prototype AlB_2_) and the CaIn_2_ and MgGa_2_ structures (space group *P63/mmc*, NO.194, two units per cell^25^,^26^); and (3) *orthorhombic*, including the SrAl_2_ structure (space group *Imma*, NO.74, four units per cell, prototype CeCu_2_^27^), another MgGa_2_ structure (space group *Pbam*, NO.55, eight units per cell^28^) and the “CaIn_2_” structure (space group *Pnma*, NO.62^20^). Many phases have already been found. At low pressure (under 160 GPa), the cubic phase (space group 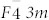, NO.216) is the most stable, while the *P63/mmc* phase is dynamically unstable and can turn into low-symmetry *Pnma*. At pressures greater than 160 GPa, the *P63/mmc* phase is more stable^2^. There is still an open question regarding whether there exist other stable phases with low energy in the low pressure zone. Therefore, further theoretical studies, such as of the crystalline structure, electronic properties and mechanical properties, are important for this potential material.

In the present work, potential crystal structures of BeB_2_ were extensively explored using *ab initio* evolutionary simulations. A new phase with *Cmcm* symmetry was uncovered. The crystal structures, phonon dispersions, mechanical properties, phase transitions, and electronic structures of this phase as well as other available phases were investigated. The band structures and density of states reveal that the new phase is metallic. Further study on the mechanical properties using three different methods was used to predict the Vickers hardness of the newly discovered *Cmcm* phase. Charge density difference maps and Mulliken population analysis, as well as COHP diagrams, were also carried out to analyze the character of the bonding between the atoms in *Cmcm* BeB_2_. The superconductivity of the new *Cmcm* phase was also investigated.

## Results

### Crystal Structure

In a previous work, several phases were found both experimentally and theoretically. However, only four structures are stable according to the calculated phonon spectra in the present work. These are cubic (space group 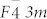, NO.216), orthorhombic (space group *Pnma*, NO.62), orthorhombic (space group *Pbam*, NO.55) and hexagonal (space group *P63/mmc*, NO.194). In the present work, a new stable phase with space group NO.63 was uncovered by performing systematic structure searches for BeB_2_. Its optimized equilibrium lattice parameters, axial ratio *c*/*a* and unit cell volume are shown in Table 1. The crystal structure information of all other known dynamical stable phases are presented in Table 2^2^,^20^. Table 1 reveals that the conventional unit cell of the new *Cmcm* phase contains twelve atoms in total with two inequivalent Wyckoff positions 4*c* and 8*f* for Be and B atoms, respectively.

**Figure 1:**
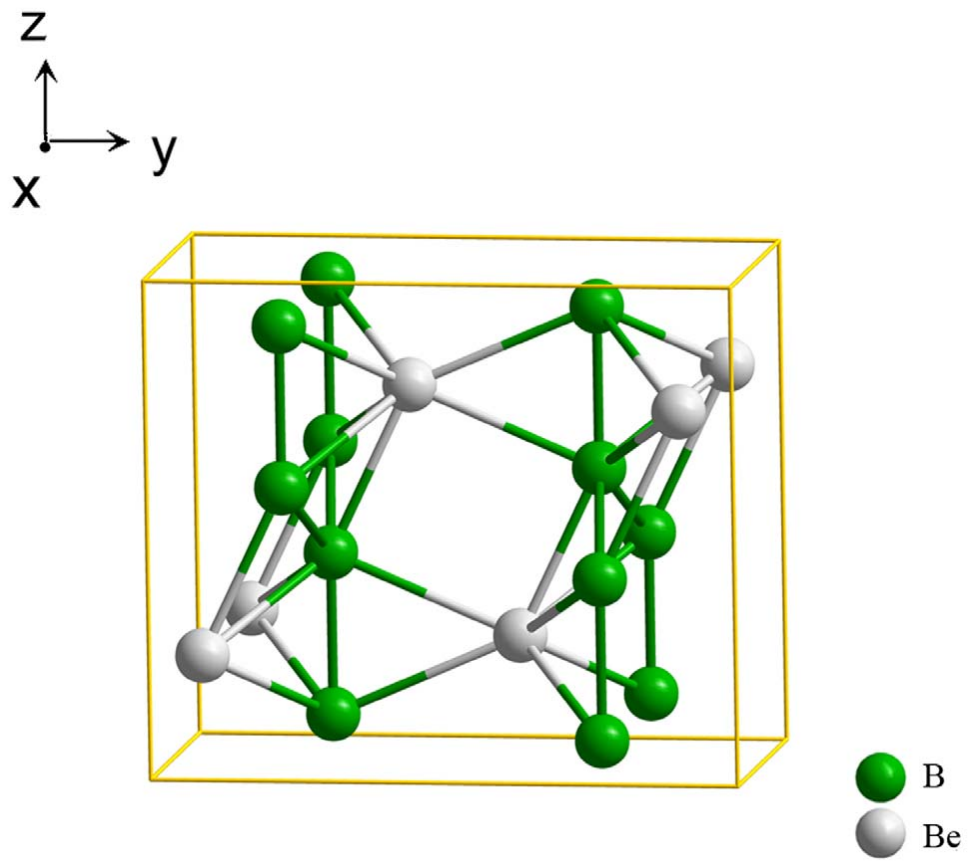
Unit cell of the crystal structure of the new *Cmcm* phase.

**Figure 2:**
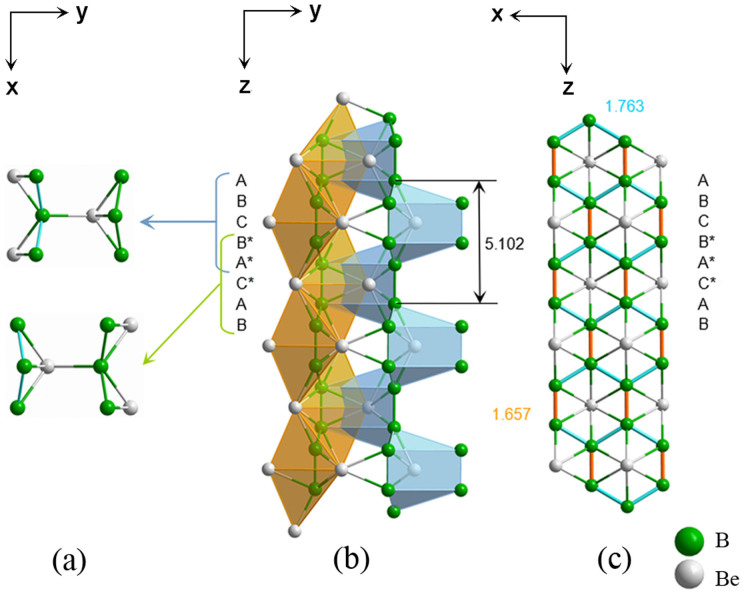
The crystal structure of the new *Cmcm* phase projected along different directions. (a) view along the direction of the *z*-axis. (b) view along the direction of the *x*-axis. (c) view along the direction of the *y-*axis.

**Figure 3:**
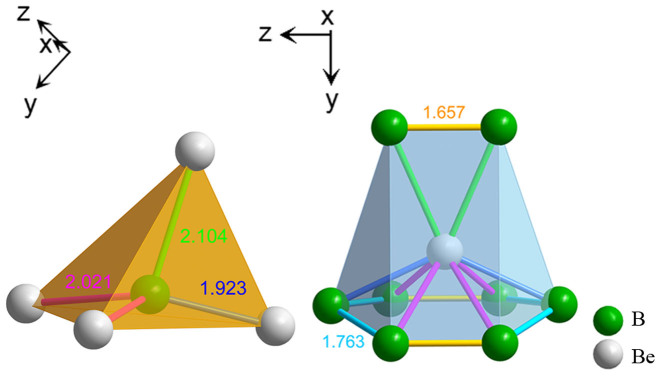
The coordination polyhedrons of the new *Cmcm* phase. (a) The coordination polyhedrons for the B atoms. (b) The coordination polyhedrons for the Be atoms.

The schematic crystal structure of the *Cmcm* phase is shown in Figures 1, 2 and 3, where yellow and blue spheres represent Be and B atoms, respectively. Figure 1 shows the unit cell, while Figure 2 shows the super-cell of the same crystal structure. Figure 2(b) is perpendicular to the *x*-axis. There are two parallel columns formed by boron-centered and beryllium-centered coordination polyhedra that are marked by green and blue colors, respectively. Each boron atom is surrounded by four beryllium atoms that form a distorted tetrahedral configuration. Eight boron atoms embrace a beryllium atom, forming a complex polyhedron composed of 3 rectangles and 6 triangles. Along the *z*-axis, the structure can be viewed as several layers, as shown in Figure 2 (b) and (c). Six boron atoms connect to each other in a hexagonal ring, where 4 atoms are coplanar (denoted as B and B*) and the other 2 atoms are out of this plane (denoted as A and A*). Be atoms form another layer (denoted as C) between the B and B* layers along the *z*-axis. The upper image of Figure 2 (a) is taken from the A, B, C, B* and A* layers, whereas the lower image is composed of the B*, A*, C*, A and B layers. The distance between two A layers is 5.120 Å. The configurations of boron-centered and beryllium-centered coordination polyhedra are displayed in Figure 3. The distances of the three types of Be-B bonds are 1.923 Å, 2.021 Å and 2.105 Å, respectively, as shown in Figure 3 (a). The Boron-Boron bond lengths are 1.763 Å (the blue bond) and 1.657 Å (the green bond), as shown in Figure 3 (b).

### Phonon Dispersion

**Figure 4:**
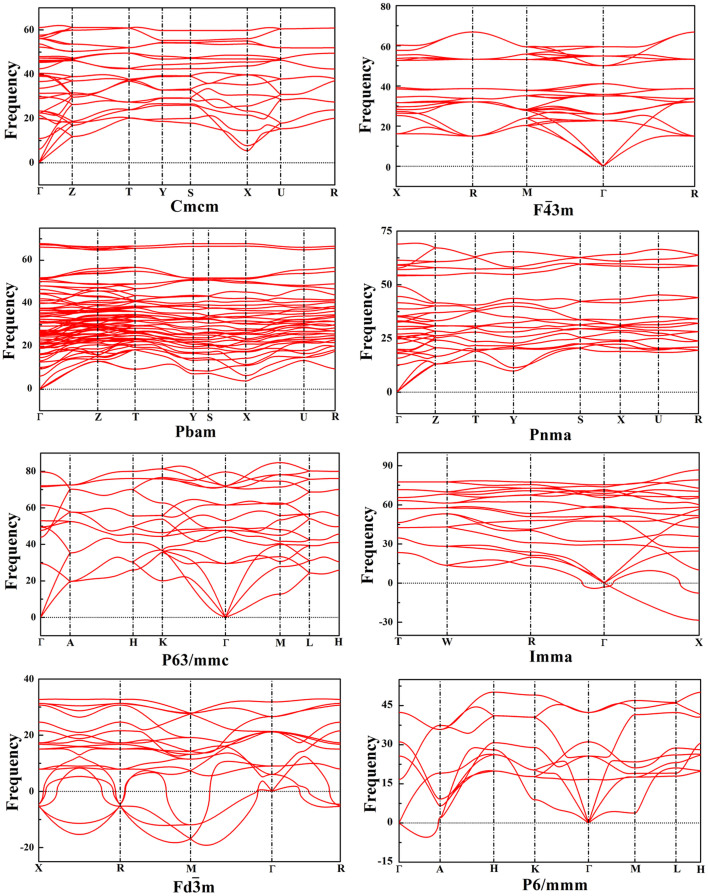
Phonon dispersion curves of available BeB_2_ phases.

The phonon dispersion curves of the new phase as well as of other previously proposed crystalline structures were calculated to determine their dynamical stability, as shown in Figure 4. The new *Cmcm* phase is dynamically stable, as there are no imaginary phonon frequencies detected in the whole Brillouin zone. For the previously proposed phases^2^,^20^, the *Pbam*, *Pnma*, 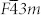 and *P63/mmc* phases are dynamically stable according to the same rule. However, the appearance of imaginary frequencies indicates that the *P6/mmm*, 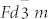 and *Imma* phases mentioned in previously published articles^2^,^20^ are dynamically unstable.

### Phase Transition

**Figure 5:**
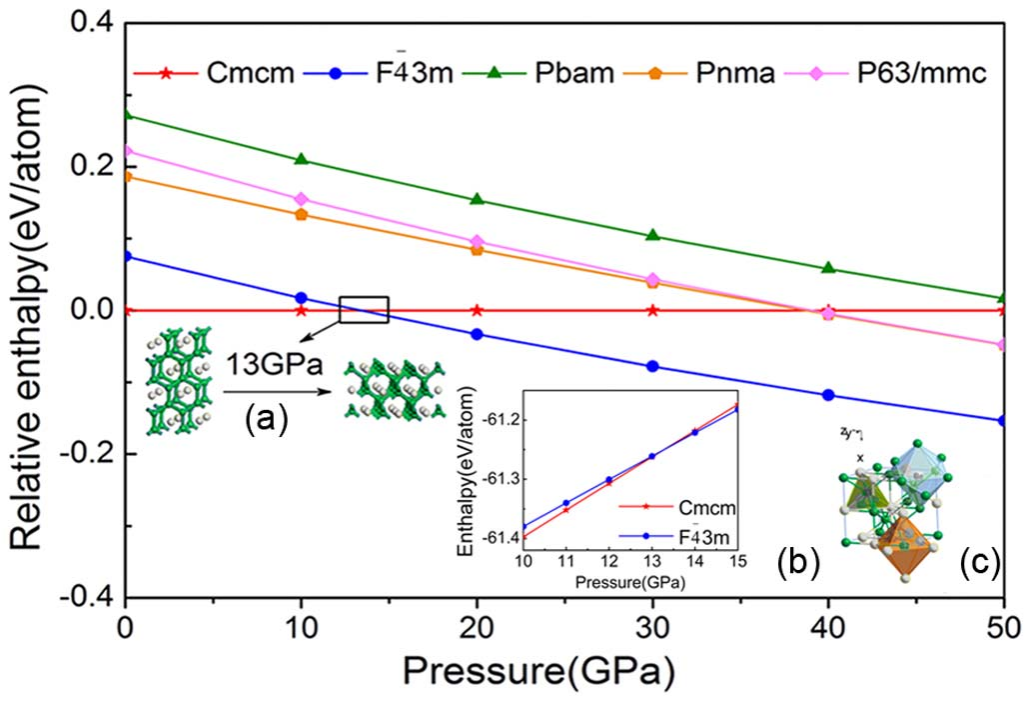
Calculated enthalpy as a function of pressure for all mechanical and dynamical stable phases of BeB_2_. (a) The *Cmcm* phase transforms into the 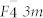 phase at a pressure of 13 GPa. (b) The coordination polyhedrons of the known 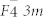 phase. The blue spheres represent B atoms, and the yellow spheres represent Be atoms.

Figure 5 gives the calculated relative enthalpy as a function of pressure for all stable phases of BeB_2_, relative to the 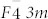 phase. The inserted figures show the stable *Cmcm* phase and the 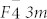 phase of BeB_2_, before and after the phase transition at 13 GPa, respectively. As mentioned before, the latter has been considered in previous works to be the most stable phase at atmospheric pressure, having a structure with a diamondoid boron network and beryllium atoms occupying the interstitial tetrahedral sites. The polyhedron representation of the 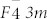 phase has also been marked, as shown in the bottom-right corner of Figure 5.

As shown in Figure 5, the enthalpy of the newly uncovered phase increases with pressure within the studied pressure range up to 50 GPa, while the enthalpies of the other three stable phases (*Pnma* phase, *Pbam* phase, *P63/mmc* phase) have an inverse trend. It can be concluded that the *Cmcm* phase is more competitive than the previously known most stable 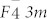 phase at pressures up to 13 GPa. When the pressure exceeds 40 GPa, the *Pnma* phase and *P63/mmc* phase have almost similar enthalpies, and both of them become more favorable than the new *Cmcm* phase. The cubic structure (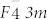 phase) continues to have the greatest stability in the pressure range from 13 GPa to 50 GPa. These results are consistent with the results previously found by Andreas Hermann *et* *al.*^2^

First-principles methods were used to calculate the formation enthalpy of the new phase *Cmcm* (No.63) at different pressures. The structural parameters of each phase are listed in Table 3. The enthalpy of formation is calculated as follows: 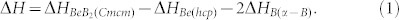
As shown in Table 4, when the pressure is 1 atm, the formation enthalpy of the *Cmcm* phase is −56 meV/atom, which is within the range of formation enthalpies (−40 meV to −125 meV) of some proposed models for BeB_2.79_ phases^20^. This may explain why the BeB_2.79_ phase with a very complex structure has been found experimentally, rather than the pure BeB_2_ phase with the simple *Cmcm* structure. It also indicates that the proposed models of BeB_2.79_ phases^20^ with enthalpies less than −56 meV/atom are more competitive. Upon comparing the formation enthalpy of the new phase (− 0.056 eV/atom) with that of the most stable known phase 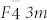 (−0.016 eV/atom), it may be observed that the former is more competitive than the latter. In short, the *Cmcm* phase is more thermodynamically stable than any other known BeB_2_ phase.

Using the same approach, the formation enthalpy of the *Cmcm* phase at 10 GPa and 15 GPa was calculated to be −39.7 meV/atom and −32.2 meV/atom, respectively. By comparing to the value at 1 atm (−56.267 meV/atom), it may be seen that the formation enthalpy value increases continuously with the pressure, indicating that increasing pressure is not beneficial for the synthesis of the *Cmcm* phase. All of these conclusions agree well with those obtained from the pressure phase diagram^2^,^20^, and the results will provide guidance for experimental work on BeB_2_ synthesis.

**Figure 6:**
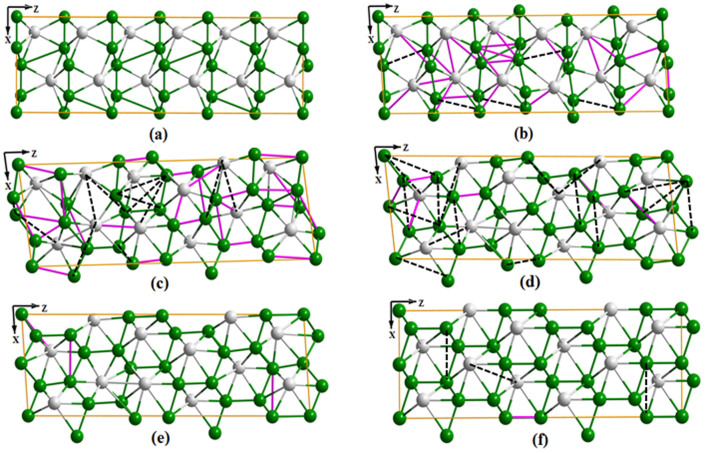
Snapshots from a dynamical trajectory collected from transition path sampling connecting (a) the *Cmcm* phase to (f) the 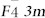 phase. Black dashed line means a bond disappears while purple solid line means a bond created compared to the previous snapshot. Boron (Beryllium) atoms are shown in green (gray) colors.

In addition, to provide more “physics” of the phase transition, we have calculated the solid-solid structural phase transitions between the *Cmcm* and 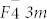 phases at 13 GPa by using the VC-NEB method^29^. Figure 6 illustrate the snapshots from a dynamical trajectory collected from transition path sampling connecting the *Cmcm* phase and the 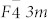 phase. The energy barrier of the transition was calculated to be 0.25 eV/atom.

## Discussion

### Mechanical Properties

Mechanical properties, including the elastic constants, bulk modulus, shear modulus, Young's modulus, Poisson's ratio, are the main bases for choosing and designing materials. Therefore, all these properties were calculated using the first-principles approach in this work. The elastic constants of all structures under the ambient pressure *C_ij_* were calculated with the strain-stress method combined with Hooke's Law, as implemented in the CASTEP code^30^. The results are listed in Table 5. In order to determine the mechanical stability of the new predictions, *C_ij_* has to satisfy the elastic stability criteria^31^.

The mechanical stability condition and elastic constants of the orthogonal structure are positive. However, the following inequalities must also be satisfied to indicate stability: 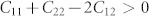

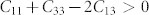

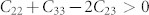

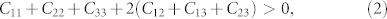


As shown in Table 5, these values all meet the mechanical stability criteria, initially confirming that the *Cmcm* phase is mechanically stable.

Based on the Voigt-Reuss-Hill approximation method^32^,^33^, we can find the appropriate bulk modulus *B* and shear modulus *G* using the elastic constants. In addition, the values of Young's modulus *E* and Poisson's ratio *σ* can be calculated using the following formula: 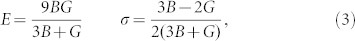
Usually, the bulk modulus *B* is used to characterize a material's resistance to volume deformation against external pressure, while the shear modulus *G* measures a material's ability to resist shear strain. Young's modulus *E* measures the resistance against longitudinal tensions. Among the 4 mechanical and dynamical stable BeB_2_ phases, the *Cmcm* phase has the lowest value of bulk modulus and the second lowest shear modulus, revealing that it has low resistance to compression and shear strain. In addition, the elastic constant *C*_22_ (282 GPa) is significantly smaller than *C*_11_ (492 GPa) and *C*_33_ (690 GPa), revealing that resistance along the *b* axis is much smaller than along the *a* and *c* axes.

In order to evaluate the ductility of the material, the *B*/*G* values were calculated and are listed in Table 5. Higher *B*/*G* values greater than 1.75 correspond to a ductile material, while values less than 1.75 correspond to a brittle material^34^. As shown in Table 5, the *B*/*G* value of the *Cmcm* phase is approximately 1.01, suggesting that it is very brittle.

Poisson's ratio reflects the strength of the covalent bond to some extent. A small Poisson's ratio (*σ* = 0.13) indicates that the *Cmcm* phase is more intensely covalently linked than the *Pbam* phase (*σ* = 0.29) and *P63/mmc* phase (*σ* = 0.18). These results show that the *Cmcm* phase is likely to be a type of potential ultra-incompressible material, despite its bulk modulus and shear modulus not being very high.

Finally, the hardness of the *Cmcm* phase was calculated in three different ways. Using a recently proposed simple empirical hardness formula *H*_v_ = 2(*G*^3^/*B*^2^)^0.585^–3^35^, the Vickers hardness of these phases was calculated. As the result shows, the *Cmcm* phase has a hardness value of 36.8 GPa, approaching the critical value of a super-hard material, 40 GPa. It also reveals that the predicted hardnesses of the *Pnma* phase and the 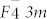 phase are approximately 41.4 GPa and 42.3 GPa, respectively, implying that both of them are potential super-hard materials. The hardness value for the *Cmcm* phase is also calculated to be 25.2 GPa using th he formula of hardness given by Artem R. Oganov^36^ (details can be found in the computational methods section).

The hardness of the new phase was also calculated by the microscopic hardness model proposed by F.M. Gao *et al.*^37^,^38^ (details can be found in the computational methods section). The calculated parameters and hardness of the *Cmcm* phase calculated by this model are listed in Table 6. It is found that the total hardness of the *Cmcm* phase is only 13.8 GPa with this method.

Chen's model gives the highest values (36.8 GPa) from these three models followed by Oganov's model (25.2 GPa) while Gao's model provides the lowest values (13.8 GPa), which may be caused by their applicability to the boron compounds and with metals. The scattered values of hardness predicted from these three different models reveal that although it has a simple crystal structure, the electronic structure and bonding characters of the newly uncovered *Cmcm* phase of BeB_2_ are quite complex, as will be analyzed below.

### Electronic Properties

**Figure 7:**
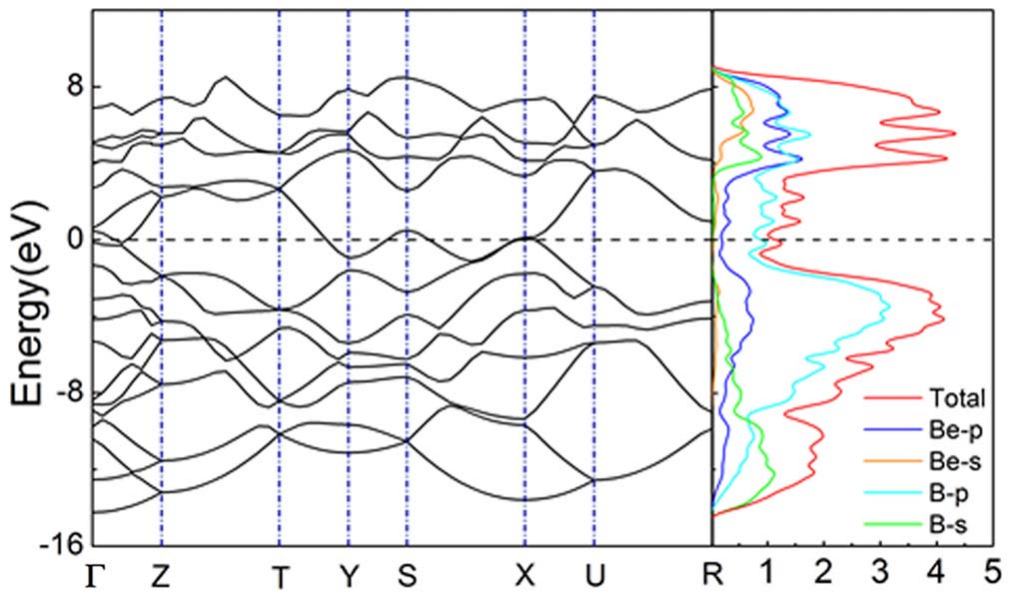
The electronic band structure and density of states for the *Cmcm* phase. The dashed transverse line denotes the Fermi level.

**Figure 8:**
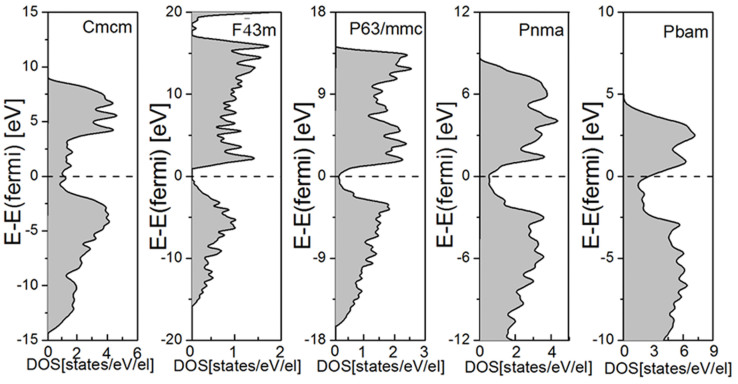
Density of states for BeB_2_ in various stable phases. From left to right: *Cmcm*, 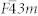, *P63/mmc*, *Pnma* and *Pbam*. The dashed lines represent the position of the Fermi level.

**Figure 9:**
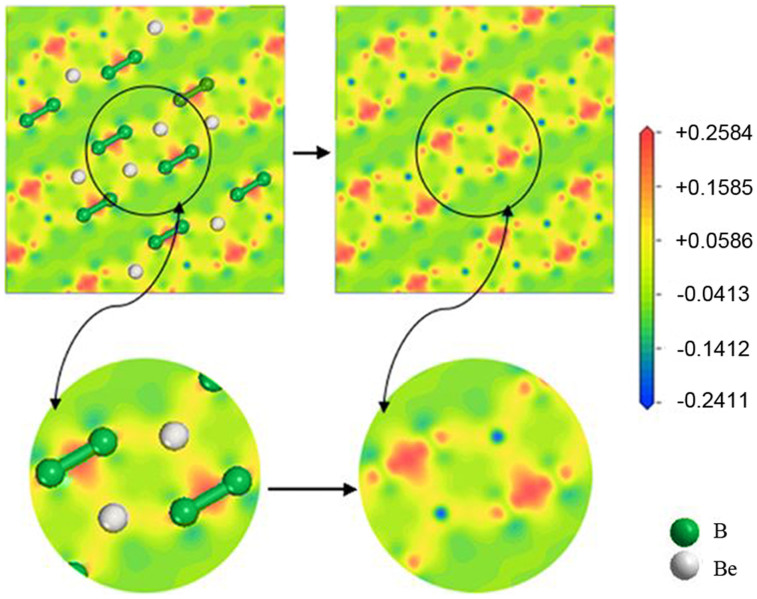
Electron density difference plot (e/Å^3^) for the *Cmcm* phase at a select slice of (100).

The electronic properties of the newly discovered phase, including energy band structures, total and partial density of states (DOS), and charge density maps, were calculated and are shown in Figure 7,8,9.

Figure 7 shows the calculated band structure along high symmetry directions, as well as the total and partial DOS of the optimized *Cmcm* structure from first-principles calculations within the GGA scheme. The overlapping of the valence bands and conduction bands around the Fermi level suggests that the new phase has a clear metallic character, which is confirmed by the finite value of total DOS at the Fermi level.

Figure 7 also plots the partial DOS of the novel *Cmcm* phase. It reveals that the total DOS of the upper part of the valence bands (from −9.5 eV to the Fermi level) is mainly contributed by the B-*p* state, while that of the conduction bands come from both the B-*p* and Be-*p* states. The Be-*s* state also contributes to the DOS of the conduction band (above 4 eV), while making a very slight contribution to that of the valence band. The B-*s* state contributes to the lower part of the valence band (less than −7.5 eV) and to the upper part of the conduction band, especially in the 4 eV to 8 eV region of the total DOS. As discussed above, there is significant hybridization of the *s* and *p* states from both Be and B in the region of 4 eV to 8 eV, implying the tendency to form covalent bonds between Be and B atoms.

Figure 8 plots the total DOS for all stable phases at ambient pressure, corresponding to the *Cmcm*, 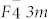, *P63/mmc*, *Pmma* and *Pbam* phases, from left to right. The dashed lines represent the position of the Fermi level. The first panel is the newly uncovered *Cmcm* phase, and the others are other known dynamically stable phases. Filling electrons at the Fermi level (approximately 1.18 states/eV/el.) shows that the new phase has an obvious metallic character, while the cubic 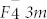 phase with a band gap of 0.95 eV indicates its semiconductor property at low and intermediate pressures^2^. Except for the 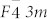 phase, all other known phases are metallic, as shown in the last three panels of Figure 8.

Figure 9 shows electron density difference maps for the *Cmcm* phase on the selected slice (100) plane. It can be seen that electrons are gathered at the positions of the B atoms and especially between Boron-Boron bonds, as indicated by the region in red in the top left panel and its enlarged bottom left panel. After removing the atoms, it is clearly seen that electrons are transferred from the Be atoms to the B atoms, as shown in the right two panels. From the electron gathering region between Boron-Boron, one can speculate that there is a strong covalent interaction.

In order to give some insight into these bonding characters, an atomic and bond Mulliken population analysis, which can provide a good way to quantitatively evaluate the charge transfer chemical bond strength of the studied system, was performed and analyzed by the CASTEP code, with the results listed in Tables 7 and 8. From Table 7, it can be clearly seen that electrons transfer from the Be atoms to the B atoms in the *Cmcm* phase, as the charges for Be and B are 0.69 and −0.34, respectively.

Now let us turn to the bond Mulliken population analysis results. A high nonzero value of overlap population indicates that there is a strong covalent character of the bond, while a small value close to zero shows that there is weak or no interaction between two related atoms and a negative value indicates that the atoms cannot form a bond^39^. From Table 8, it may be seen that the bond population ranges from 0.20 to 1.63 for the *Cmcm* phase. The maximum number, 1.63, exists between Boron-Boron bonds, indicating their strong covalent character. However, the Be-B bond has very weak covalence, as its Mulliken population is only 0.20. The population of Be-Be is −0.24, suggesting that there are no bonds forming. All of these Mulliken population results are consistent with the conclusions drawn from the electron density difference maps. As discussed in the mechanical properties section of this work, the hardness of the *Cmcm* phase may be a result of the high population value of the Boron-Boron bonds.

**Figure 10:**
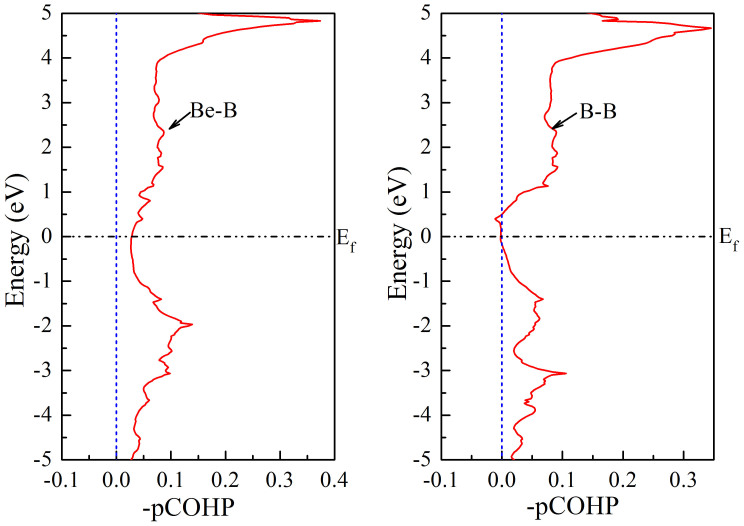
The crystal orbital Hamilton population (COHP) analysis of the bonding interactions in BeB_2_ based on plane-wave calculations using the newly introduced pCOHP method. (a) for the Be-B bond. (b) for the B-B bond. All energies are shown relative to the Fermi level E_f_.

Some techniques such as the crystal orbital overlap population (COOP)^40^,^41^ and its analogous crystal orbital Hamilton population (COHP)^42^,^43^ can provide a straightforward view of orbital-pair interactions; based on these techniques, it is possible to analyze and interpret the bonding situation in solid-state materials. To elucidate the bonding situations in this new BeB_2_ phase, we performed crystal orbital Hamilton population (COHP) analysis, which partitions the band structure energy (in term of the orbital pair contributions) into bonding, nonbonding and anti-bonding energy regions within a specified energy range. Figure 10 shows the resulting –pCOHP as a function of energy for the new phase. Positive values of –pCOHP describe bonding energy regions, whereas negative values describe anti-bonding energy regions. As seen in the COHP diagrams in Figure 10, there appear to be obvious Be-B bonding states at the Fermi level, while that is not the case for the B-B combination, indicating that the interactions between Be-B bonds in the unit cell are stronger than those of B-B bonds, despite the fact that a single Be-B bond is weaker than a single B-B bond, as shown by the Mulliken population. Above or below the Fermi level (in the range of −6 to 6 eV), the COHP plots of the Be-B and B-B combinations are clearly dominated by bonding states, which shows that the new phase has a favorable stability performance.

### Superconductivity Properties

**Figure 11:**
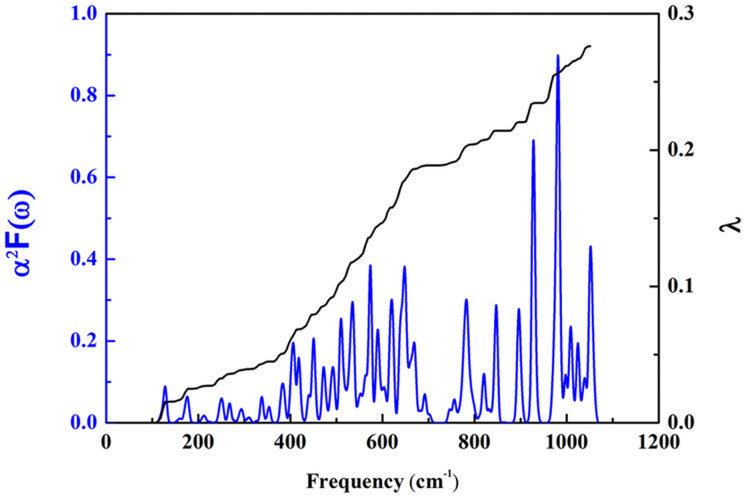
The calculated spectral function *α*^*2*^*F*(*ω*) and integrated *λ*(*ω*) of the *Cmcm* phase.

Encouraged by the relatively simple binary MgB_2_ having a superconductivity transition temperature of 39 K^44^ and the controversy regarding the reported superconductivity properties of BeB_2_ and BeB_2.75_^19^,^21^, we also calculated the superconductivity properties of the *Cmcm* phase of BeB_2_. The *T*_c_ can be estimated from the Allen-Dynes modified McMillan equation^45^, 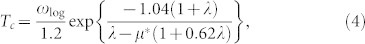
which has been found to be highly accurate for materials with an EPC constant λ<1.5^46^, where *ω_log_* is the logarithmic average of the phonon frequency and μ* is the effective Coulomb repulsion and was assumed to be constant at 0.1. The calculated spectral function *α*^*2*^*F*(*ω*) and integrated *λ*(*ω*) of the *Cmcm* phase are plotted in Figure 11. Our results reveal that the *Cmcm* phase exhibits fairly low superconductivity properties, with a *T*_c_ of only 0.1 K. These results shed light on the controversy regarding the reported superconductivity properties of BeB_2_ or BeB_2.75_. The synthesized sample may contain both BeB_2_ and BeB_2.75_ phases. When the BeB_2_ phase dominates, an absence of superconductivity would be observed, as shown in Ref. [21], while when the BeB_2.75_ phase dominates, superconductivity appears.

## Methods

*Ab initio* evolutionary simulations were run using the USPEX (Universal Structure Predictor: Evolutionary Xtallography) code^47^,^48^,^49^.

The USPEX code depends on VASP (Vienna *ab initio* simulation package)^50^ to achieve global optimization to calculate the enthalpy of crystal structures and explore the lowest enthalpy phase of a given elemental composition. Here, we used the USPEX code to search for stable compounds and structures with a fixed chemical composition of Be_n_B_2n_ (n = 1 to 5); the *Cmcm* phase comes from the 25th structure with the stoichiometry of Be_2_B_4_. During the structure search, USPEX selects a whole range of 50 generations to calculate, with each generation containing 50 individuals. The settings used for the variation operators are as follows: 60% of each generation was used to produce the next generation by heredity, 20% comes from soft mutations, 10% is produced randomly from space groups, and the rest is produced through lattice mutations. The minimum length of any lattice vector was defined as 2.0 Å. The cutoff for USPEX relaxation and the k-points for resolution were 318 eV and 2π × 0.02 Å^−1^, respectively.

First-principles^51^ calculations were carried out using the density functional theory (DFT) approach by applying a generalized gradient approximation (GGA) for the exchange correlation functional^52^,^53^. We applied the Ultrasoft pseudo-potential introduced by Vanderbilt^54^, and the k-point samplings in the Brillouin zone were performed using the Monkhorst-Pack Scheme. The convergence tests used a kinetic energy cutoff of 600 eV and a k-point of 13 × 7 × 8 for the predicted *Cmcm* phase in the geometry optimization calculations. The self-consistent convergence of the total energy was 5 × 10^−7^ eV/atom, the maximum force on each atom was below 0.01 eV/Å and the maximum atomic displacement was below 5 × 10^−4^ Å. The phonon dispersion curves were plotted using the super-cell calculation method^55^ applied in the Phonopy program^56^. The calculation of the elastic constant and Mulliken overlap populations was carried out using the CASTEP code^57^. From the calculated elastic constants *C_ij_*, the polycrystalline corresponding bulk modulus *B* and shear modulus *G* were calculated using the Voigt-Reuss-Hill approximation^32^,^33^. In addition, Young's modulus *E* and Poisson's ratio *σ* were obtained by the equations *E* = (9*G·B*)/(3*B* + *G*), and *σ* = (3*B*−2*G*)/(6*B* + 2*G*), respectively.

In addition, we used three different methods to calculate hardness. These three methods were proposed by Xing Qiu Chen^35^, Artem R. Oganov^36^ and Faming Gao *et al.*^37^,^38^, respectively. The description of Chen's model can be found in the text. To use the formula of hardness given by Artem R. Oganov^36^, we need to use the structure file POSCAR (which must contain an element symbol line) and set the parameters for goodBonds, valence and valence electrons. For main group elements, only the outermost electrons are considered as valence electrons under normal circumstances. Hence, the numbers of valence electrons for Be and B atoms are 2 and 3.

Details of Gao's model are described below. The Vickers hardness of complex crystals can be calculated by a geometric average of all bonds as follows, 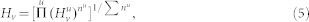
For the *Cmcm* phase, the total hardness is given by 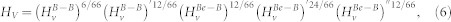
Because no *d*-orbital valence electrons are involved in the chemical bonds, the hardness of each bond for *Cmcm* phase can be expressed by: 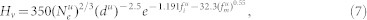
where *d^u^* is the length of the bond, 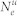 is the valence electron density (which can be calculated by 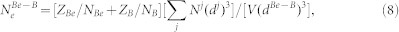

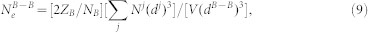
where *Z_Be_* and *Z_B_* are the valence electron numbers of the Be and B atoms constructing Be-B or B-B bonds, *N_Be_* and *N_B_* are the nearest coordination numbers of the Be and B atoms, *N_j_* is the number of j bond in the unit cell, and *V* is the volume of the unit cell) and 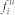
is the Phillips ionicity of the bond. According to the generalized ionicity scale, the Phillips ionicity can be obtained from the following formula, 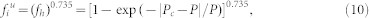
where *f_h_* is the population ionicity scale of the chemical bond, *p* is the overlap population of the bonds, and *p_c_* is the overlap population of the bonds in a specified pure covalent crystal (here 0.57 is adopted).

For the complex crystal compounds, we considered three effects on hardness: the covalent component, the ionic component and the small metallic component. First, we defined a factor of metallicity *f_m_* as *n_m_*/*n_e_* for a simple-structured compound, where *n_m_* and *n_e_* are the numbers of electrons that can be excited at the ambient temperature and the total number of valence electrons in the unit cell, respectively. According to the electronic Fermi liquid theory, the thermally excited electron number *n_m_* can be described by the product of *D_F_* and the energy width *kT*, where *k* is the Boltzmann constant and *T* is the temperature. At the ambient temperature, *kT* is equal to 0.026 eV. Therefore, *f_m_* can be written as: 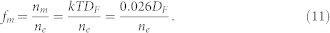
When the chemical bonds of a crystal are greater than or equal to two, we refer to it as a complex crystal. For the metallicity of complex crystals, the *f_m_* can be calculated by 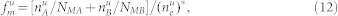
where 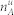 (or 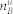) is the thermally excited electron number of Be or B atoms in the *u*-type bond, *N_MA_* (or *N_MB_*) is the number of chemical bonds with a metallic component around Be or B atoms, and 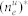 is the number of valence electrons per *u*-type bond.

To elucidate the bonding information in this new phase, we adopted a variant of the familiar COHP approach that stems from a PW calculation and was dubbed “projected COHP” (pCOHP)^58^,^59^. In this approach, all of the projection and analytic methods are implemented in a standalone computer program that processes PAW parameters and self-consistent results from VASP.

The calculation of the electron-phonon coupling (EPC) parameter λ was performed using the pseudo-potential plane-wave method within the density functional perturbation theory (DFPT)^60^ as implemented in the Quantum Espresso package^61^ by using Von Barth-Car type norm-conserving pseudo-potential with cutoff energies of 80 and 320 Ry for the wave functions and the charge density, respectively. A 7 × 4 × 4 *q*-point mesh in the first Brillouin zone was used in the EPC calculation.

## Conclusions

In summary, a new stable phase of BeB_2_ with the space group of *Cmcm* was discovered by using *ab initio* evolutionary simulations. The *Cmcm* phase has a lower enthalpy than any previously proposed phase. The new structure is mechanically and dynamically stable, as determined by checking the calculated elastic constants and phonon dispersions, while several previously proposed phases (cubic: 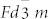; hexagonal: *P6/mmm*; orthorhombic: *Imma*) were found to be dynamically unstable. The *Cmcm* phase may transform to the cubic 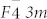 phase when the pressure exceeds 13 GPa. The calculated electronic band structure and density of state suggest that the uncovered new phase is metallic. Scattered hardness values calculated from three models suggest the complex electronic and bonding features of the *Cmcm* phase. The charge density difference maps and the Mulliken population analysis reveal that there are strong covalent interactions between the B atoms. The COHP diagrams show that the total interaction of Be-B bonds is stronger than that of B-B bonds. The *Cmcm* phase exhibits fairly low superconductivity properties, with a calculated T_c_ of approximately 0.1 K. The current theoretical predictions will most likely promote further experimental and theoretical investigation on the Be-B system.

## Author Contributions

C.Z.F. conceived the idea. Y.J. performed the *ab initio* evolutionary simulations and DFT calculations. Y.J. and J.L. carried out the hardness predictions. J.L. did the COHP analysis. X.D. performed the superconductivity properties calculations. C.Z.F.and Y.J. wrote the manuscript with contributions from all.

## Figures and Tables

**Table 1:** Optimized structual data for the new phase of BeB_2_

Space group	*a*	*b*	*c*	*c*/*a*	*α*	*β*	*γ*	*ρ*	*V*
*Cmcm*	2.988	6.028	5.120	1.714	90.0	90.0	90.0	2.206	23.06
atom		position		*x*		*y*		*z*	
Be		4*c*		0.50		0.4090		0.7500	
B		8*f*		1.00		0.2299		0.5882	

**Table 2:** Optimized structural data for other known phases of BeB_2_

Space group	*a*	*b*	*c*	*c/a*	*α*	*β*	*γ*	*ρ*	*V*
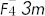	4.311	4.311	4.311	1.000	90.0	90.0	90.0	2.539	20.03
*Pnma*	5.064	3.035	5.285	1.044	90.0	90.0	90.0	2.505	20.31
*Pbam*	4.656	11.316	2.997	0.644	90.0	90.0	90.0	2.577	19.74
*P63/mmc*	3.004	3.004	5.014	1.669	90.0	90.0	120.0	2.597	19.59

**Table 3:** The parameters for structure used in the formation enthalpy calculation

phase	Space group	No.	*a*	*b*	*c*	*α*	*β*	*γ*	*ρ*
BeB_2_	*Cmcm*	63	2.996	6.0428	5.1017	90	90	90	2.2029
Be	*P63/mmc*	194	2.249	2.249	3.569	90	90	120	1.9150
B	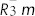	166	5.036	5.036	5.036	58.076	58.076	58.076	2.496

**Table 4:** Formation enthalpy for BeB_2_ phases at P = 1 atm

Space group	*P6/mmm*	*Pnma*	*Pbam*	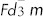	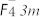	*P63/mmc*	*Cmcm*
Δ*H_f_* (eV/atom)	+0.404	+0.138	+0.238	+0.853	+0.019	+0.166	-0.056
Δ*H_f_^’^* (eV/atom)	+0.120	+0.099	+0.179	+0.687	−0.016	——	——

**Table 5:** Calculated values of elastic constants *C*_ij_ (in GPa), Bulk modulus *B* (in GPa), Shear modulus *G* (in GPa), Young's modulus *E* (in GPa), Poisson's Ratio *σ*, *B*/*G* ratio, and Vick's Hardness (in GPa) calculated by Xing Qiu Chen's^34^ formula for stable phases of BeB_2_ at 0 GPa and 0 K

	*Cmcm*	*Pbam*	*Pnma*	*P63/mmc*	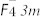
*C_11_*	492	477	458	515	482
*C_22_*	282	555	519	—	—
*C_33_*	690	530	482	389	—
*C_44_*	96	22	228	160	220
*C_55_*	263	25	146	—	—
*C_66_*	109	224	116	—	—
*C_12_*	35	83	0.4	128	81
*C_13_*	34	36	-0.5	80	—
*C_23_*	7	26	61	—	—
*B*	169	205	175	218	215
*G*	168	99	183	177	212
*E*	379	256	407	418	479
*σ*	0.13	0.29	0.11	0.18	0.13
*B/G*	1.01	2.07	0.96	1.23	1.01
*H_v_*	36.8	9.5	41.4	29.4	42.3

**Table 6:** Parameters related to hardness and the value of Vickers hardness for *Cmcm* phase and the total hardness is 13.8 GPa

bond		*d (Å)*	*N*	*p*	*N_e_^u^ (Å^−3^)*	*p_c_*	*f_i_*	*f_m_ (10^−3^)*	*H_v_ (GPa)*
B-B	B-B	1.657	6	0.84	0.998	0.57	0.387	0.953	30.9
	B-B′	1.763	12	1.63	0.828		0.581	0.953	18.5
Be-B	Be-B	1.923	12	0.24	0.505		0.807	0.889	8.4
	Be-B′	2.021	24	0.49	0.435		0.248	0.889	13.1
	Be-B″	2.105	12	0.20	0.385		0.882	0.889	12.4

**Table 7:** Atomic Mulliken populations for *Cmcm* phase

Cmcm	*s*	*p*	Total	Charge(*e*)
Be1-Be4	0.10	1.21	1.31	0.69
B1-B8	0.84	2.50	3.34	−0.34

**Table 8:** Bond populations and bond lengths (Å) for *Cmcm* phase

phase	Bond	Population	Length	Bond	Population	Length
*Cmcm*	B1-B5	0.84	1.657	Be-Be	−0.24	2.785
	B1-B8	1.63	1.763	B3-B5	−0.10	2.915
	Be4-B1	0.24	1.923	B2-B3	−0.48	2.974
	Be2-B2	0.49	2.021			
	Be3-B4	0.20	2.104			
